# How does job mobility relate to work commitment among rural healthcare workers? a cross-sectional study in western China

**DOI:** 10.1186/s12913-021-07166-w

**Published:** 2021-10-20

**Authors:** Jinlin Liu, Ying Mao, Bin Zhu

**Affiliations:** 1 School of Public Policy and Administration, Northwestern Polytechnical University, Xi’an, Shaanxi 710072 China; 2 School of Public Policy and Administration, Xi’an Jiaotong University, Xi’an, Shaanxi 710049 China; 3 School of Public Health and Emergency Management, Southern University of Science and Technology, Shenzhen, Guangdong 518055 China

**Keywords:** Job mobility, Work commitment, Healthcare worker, Rural China

## Abstract

**Background:**

Rural healthcare workers (RHWs) are the core of the rural health system. The antecedents of turnover of RHWs have been well studied, but little is known about the consequences of job mobilities among RWHs. This study aimed to identify the association between job mobility and the work commitment of RHWs in China.

**Methods:**

Based on a three-stage random sampling method, a cross-sectional survey was conducted in 11 western provinces in China. A total of 3783 RHWs, consisting of 2245 doctors and 1538 nurses, were included in our study. Confirmatory factor analysis, Pearson’s chi-squared test, one-way ANOVA, linear regression analysis, and binary logistic regression analysis were performed for data analyses.

**Results:**

46.3% of RHWs reported the experience of job mobility in the past. Work commitment of RHWs was not very high; specifically, the mean scores of pride in, concern for, and dedication to work were 3.54, 3.81, and 3.61 (out of a maximum of 5), respectively, and 29.9% presented turnover intent. RHWs’ overall experience of job mobility in the past was significantly associated with an increased odds of having the turnover intent. With respect to the last job mobilities of RHWs, the last job changes that occurred in the last 3 years, especially these lateral (i.e., job changes between two healthcare institutions at the same hierarchical level) and upward (i.e., job changes from a healthcare institution at a lower hierarchical level to current institution) mobilities, were significantly associated with a high level of work commitment (i.e., pride in, concern for, and dedication to work) among RHWs. However, the lateral mobilities in the last four to 5 years and the downward mobilities (i.e., job changes from a healthcare institution at a higher hierarchical level to current institution) 6 years ago or more significantly increased the odds of having turnover intent among RHWs, and RHWs whose last job changes were other mobilities (i.e., job changes from a non-healthcare institution to a healthcare institution) in the last four to 5 years reported had a significantly low level of pride in and concern for work and an increased odds of having the turnover intent.

**Conclusions:**

The study suggests that the overall experience of job mobility in the past is a threat to RHWs’ work commitment to their current healthcare institutions. The honeymoon-hangover pattern exists in the association between a single job change and RHWs’ work commitment. Managers of rural healthcare institutions should pay more attention to these RHWs with the experience of job mobility to enhance their work commitment.

**Supplementary Information:**

The online version contains supplementary material available at 10.1186/s12913-021-07166-w.

## Background

The health workforce is the core of a health system. In the context of transition from the Millennium Development Goals to the Sustainable Development Goals (SDGs), a sufficient and qualified health workforce is essential to achieve the health-related SDGs [1, 2]. However, shortage and misdistribution of qualified health workforce are still global concerns affecting nearly all countries, especially the remote and rural areas [3]. In response to the challenges of health workforce in remote and rural areas, the World Health Organization (WHO) has proposed a series of global policy recommendations to improve attraction, recruitment, and retention of healthcare workers in remote and rural areas [4]. Considering the difficulty of attraction and recruitment of health workforce in remote and rural areas, improving the retention of the existing health workforce should be given priority in these areas. Currently, a great deal of academic attention has been paid to this issue. Most research has identified the influencing factors of turnover behavior or intent of healthcare workers in remote and rural areas; however, consequences of turnover of the health workforce in remote and rural areas have been generally ignored. Some studies have examined the effects of work-related and personal attitudes such as work commitment, job satisfaction, and work stress on turnover intent of healthcare workers in rural areas [3, 5], but rarely have the effects of the real job mobility of RHWs on these attitudes been studied.

Work commitment, i.e., employee commitment or organizational commitment, is important for an organization, as its success or failure is closely related to its employees’ motivation and effort that are often the product of the work commitment of employees [6, 7]. It’s defined by Loscocco as the relative importance of work to one’s sense of self [8]. Much evidence has been found about the impact of work commitment of healthcare workers on both individual and organizational performance. For example, Yang et al. found that work commitment of healthcare workers was significantly inversely associated with their presenteeism [9]; Baird et al. reported a positive effect of the level of work commitment of healthcare workers on both patient care and operational effectiveness in hospitals [10]; and Attia et al. [11] and Horwitz et al. [12] identified the significant associations of work commitment of healthcare workers with caring efficacy and patient safety culture, respectively. However, very few studies are found to analyze the work commitment of RHWs [5, 7]. Meanwhile, given that job change is not uncommon among healthcare workers in China, for example, according to the 2019 research report of career dynamics of doctors in China [13], 37% of 1212 participated doctors reported the experience of external job mobility in the past, it is important to understand how they respond to their new jobs after the job mobility, especially their work commitment.

Some studies, though few, have investigated the association of job change with work commitment among employees in other industries [14–19]. Job mobility can be studied by the overall experience of job change in the past or be measured with respect to a single job move [16]. Our study covered both aspects; specifically, we would examine the relationship of overall experience of job mobility with work commitment, as well as a single job move. Meanwhile, our study only focused on the external job mobility of RHWs. In terms of the overall experience of job mobility, both Kondratuk et al. [16] and Murrell et al. [19] found that the experience of external job mobility in employees’ career histories was significantly negatively associated with their work commitment to current organization. From the perspective of career attitudes, Feldman et al. [20, 21] thought that among the defining characteristics of careerist attitudes were the belief that it was sometimes necessary to promote one’s own career advancement, even at the expense of organizational goals, and that commitment to an organization was unlikely to be rewarded; besides, others reported that boundaryless and protean career attitudes (i.e., organizational mobility preference) were significantly negatively correlated to employees’ work commitment [22, 23]. Based on the above research, the following hypothesis was proposed:

- Hypothesis 1. The experience of job mobility will be negatively associated with work commitment with respect to RHWs’ current healthcare institutions.

With respect to the single job move, we would assess the association of the last job mobility of RHWs with their work commitment. Due to the cross-sectional design of our study, we could not compare the changes of work commitment before and after the last job change. However, as several studies found that work commitment were quite stable over time for non-job movers [24–26], we could analyze the associations of different times of RHWs’ last job mobilities with their work commitment, as well as different types of the last job mobilities, by comparing with RHWs without job changes. For the time of the last job mobility, similar findings were reported by Swaen et al. [17], Kondratuk et al. [16], and Equeter et al. [14] that the external mobility in the past year, in the last 18 months, and in the last 3 years, respectively, could significantly improve employees’ work commitment in the new organization relative to the former organization and to other non-job movers; however, Kalleberg et al. found that external mobility in the last 5 years significantly lower the work commitment of employees in their new organizations relative to the former workplace [18]. These above findings, as well as the evidence of negative association between overall experience of external job mobility and work commitment, brought us to the honeymoon-hangover effect found by Boswell et al. [27, 28], and they reported that job satisfaction would reach a peak following job mobility and decrease thereafter. The honeymoon-hangover pattern might also exist in the associations of different times of RHWs’ last job mobilities with their work commitment. Accordingly, the following hypotheses were made:

- Hypothesis 2a. The last job mobility that occurred over relatively recent periods of time would relate positively to work commitment.
- Hypothesis 2b. The positive association of last job mobility with work commitment would weaken or even become negative after years working for the same institution.

As for the type of job mobility, we thought that different types of job changes would lead to different changes in RHWs’ working and living conditions, hence the diverse potential impacts on work commitment; however, due to a paucity of related research assessing the impact of different types of job mobilities on work commitment, no formal hypothesis could be made for these dimensions of different types, and we could only propose a general hypothesis as below.

- Hypothesis 3. The association between RHWs’ last job mobilities and their work commitment would differ by different types of these job changes.

## Methods

### Study design

The cross-sectional study was a part of a large collaborative research project that was jointly supported by the China Medical Board and the WHO [3, 7, 29–33]. The study was conducted in 11 western provinces in China, i.e., Gansu, Guangxi, Kweichow, Inner Mongolia, Ningxia, Qinghai, Shaanxi, Sichuan, Tibet, Xinjiang, and Yunnan. 11 provincial steering committees consisting of researchers from local universities were set up to implement surveys in each province [7]. The cross-sectional survey related to this study was carried out simultaneously in all 11 provinces from June to September 2013. Although the dataset was currently somewhat out of date, to our knowledge, it was the first and only one that covered rural areas in 11 western provinces in China. Besides, as there has been little progress in occupational situation of rural healthcare workforce in China’s health system, our data could still be used to find some valuable results. The provincial steering committee in Shaanxi designed the original questionnaire. Then, it was validated by research teams in other 10 provinces by group discussion and small-scale pre-surveys, and revised and finalized under the agreement of all research teams. Two experts from the WHO provided technical support for the study design. The paper version of questionnaire was used during the formal survey and was filled in by RHWs themselves anonymously. Informed consent was obtained from all subjects involved in the study.

### Setting and participants

The survey was conducted among healthcare workers in rural healthcare institutions that could provide clinical services. As defined in the China Health Statistical Yearbook, in China, the county is regarded as a rural area in a broad sense [7, 34]. According to the original study design [3, 7], the RHWs included in the survey were doctors, nurses, pharmacists, and so on. Healthcare managers and support workers were excluded. Two levels of rural healthcare institutions, including township level and county level, were involved in the survey.

A three-stage random sampling method was carried out. Under consideration of the study budget and survey duration, the sample size of RHWs was set by the PI (i.e., principal investigator), all co-PIs, and two WHO experts. The detailed sampling strategy has been reported in our previous papers [3, 7]. Specifically, in each province, according to the gross domestic product per capita ranking of all countries, three of them, that is, poor, medium, and rich counties, were first selected randomly. Second, each county in China generally has several township-level healthcare institutions, i.e., township healthcare center (THC), and four county-level healthcare institutions, that is, one center for disease control and prevention (CDC), one traditional Chinese medical hospital (TCMH), one maternity and child healthcare hospital (MCHH), and one county general hospital (CGH), so we randomly selected three THCs (if available) and invited all four county-level health institutions to participate in the survey. Third, as the number of RHWs differed across different types of health institutions, we invited all RHWs in each THC and randomly invited 30 RHWs (if available), 50 (if available), 50 (if available), and 50 (if available) in each CDC, TCMH, MCHH, and CGH. Finally, approximately 6000 RHWs were selected, and 5584 were willing to participate in the survey and completed the questionnaires [3, 7]. Furthermore, considering the objective of this study and that it focused on rural doctors and nurses, 3783 RHWs, including 2245 rural doctors and 1538 rural nurses, were extracted from the original dataset and included in this study.

### Variables

According to the objective of this study, we only extracted the relevant variables from the original questionnaire.

The dependent variable was the work commitment of RHWs, which has been conceptualized and measured in various ways [35]. Generally, it includes affective commitment, continuance commitment, and normative commitment. Our study focused on the affective commitment and its measurement has been introduced in detail in our previous paper [7]. Specifically, 10 questions were used to measure the work commitment which included a dichotomous question and a 5-point Likert scale with nine questions. The dichotomous question used to determine the turnover intent of RHWs was ‘do you have the intent to leave your current location in the next year,’ answered with either yes or no. For the other nine questions, participants were asked to rate their perceived degree on a scale of one (strongly disagree) to five (strongly agree), and the exploratory factor analysis has been conducted to induce dimension reduction and extract subdomains of work commitment in our previous paper [7]. Because the final number of participants in this study after data cleaning was different from that in our previous paper, we conducted the confirmatory factor analysis (CFA) to induce dimension reduction. The value of Kaiser-Meyer-Olkin was 0.930, and the *p*-value of Bartlett’s test of sphericity was less than 0.001, indicating acceptable construct validity. The results of three subdomains extracted based on the CFA were consistent with those in our previous paper [7], which consisted of pride (i.e., having pride in work), concern (i.e., being concerned for work), and dedication (i.e., being dedicated to work). The score of each subdomain was further calculated based on the composite ratings by averaging the responses to each question within these subdomains, and a high score of each subdomain reflected a high-level work commitment of RHWs. Finally, the work commitment of RHWs consisted of four subfactors, including three continuous variables (i.e., pride in, concern for, and dedication to work) and one binary variable (i.e., turnover intent).

The independent variable was the job mobility of RHWs. Participants were first asked to reply to a question as ‘do you have the experience of job mobility in the past?’ answered with either yes or no. As mentioned before, in our study, job mobility was defined as job changes of RHWs between different institutions, i.e., external mobility. And if the answer was yes, participants were further asked to reply to two questions about their last job mobilities. One question was about the time of the last job mobility including three groups, i.e., ≤3 years, 4–5 years, and ≥ 6 years, which referred to RHWs’ job changes in the last 3 years, in the last four to 5 years, and 6 years ago or more. Another question was about the type of the last job mobility. In China, healthcare institutions were divided into three levels, including the tertiary, secondary, and primary healthcare institutions. Based on this, four types of RHWs’ last job mobilities were set, including lateral mobility (referring to job changes between two healthcare institutions at the same hierarchical level), upward mobility (referring to job changes from a healthcare institution at a lower hierarchical level to current institution), downward mobility (referring to job changes from a healthcare institution at a higher hierarchical level to current institution), and other mobility (referring to job changes from a non-healthcare institution to current institution). In addition to the above two aspects, we did not consider whether RHWs were promoted or demoted in their last job mobilities.

In addition, we introduced nine sociodemographic characteristics of the RHWs as the controlled variables, which included: (1) type of profession with two groups, i.e., doctor and nurse; (2) gender with two groups, i.e., female and male; (3) age with three groups, i.e., ≤29 years, 30–39 years, and ≥ 40 years; (4) marriage with two groups, i.e., unmarried (never married) and married (ever-married); (5) education with three groups, i.e., ≤technical secondary school, medical college, and ≥ bachelor’s degree; (6) technical title with three groups, i.e., primary (equal to the medical assistant or resident physician), intermediate (equal to the attending physician), and senior (equal to the associate chief or chief physician); (7) income per month with three groups, i.e., ≤2000 Yuan, 2001–3000 Yuan, and ≥ 3001 Yuan; (8) administrative duty with two groups, i.e., no and yes; and (9) type of healthcare institution with two groups, i.e., township healthcare center and county-level healthcare institution.

### Statistical analysis

All categorical variables were displayed by counts and percentages. Continuous variables related to the work commitment of RHWs were presented by mean scores and standard deviations. Pearson’s chi-squared tests were conducted to identify differences in the percentages of socio-demographic characteristics and work commitment (i.e., turnover intent) between RHWs with and without experience of the job mobility in the past. One-way ANOVA (i.e., analysis of variance) was applied to assess differences in the mean scores of pride in, concern for, and dedication to work between RHWs with and without experience of the job mobility. *P-*values were reported.

In addition, we constructed multivariate linear regression models and binary logistic regression models to analyze the association of the job mobility of RHWs with their work commitment. Three subfactors of work commitment of RHWs, i.e., pride in, concern for, and dedication to work, were set as dependent variables in three linear regression models separately. Meanwhile, binary logistic regression models were set up with the turnover intent as the dependent variable. In each of these models, RHWs’ overall experience of job mobility, the time of the last job mobility, and the type of the last job mobility were set as independent variables successively. Besides, by generating a new variable, we further analyzed the interactive association of the time and type of RHWs’ last job mobilities with their work commitment. All nine controlled variables mentioned above were adjusted in these models. When performing linear regression analyses, all categorical variables were converted to dummy variables. The *b* (regression coefficient), *t* value of *b*, and *p*-value were reported for linear regression analyses. The odds ratio (OR), 95% confidential interval (CI), and *p*-value were reported when performing binary logistic regression analyses. A *p*-value < 0.05 was considered to be significant in the study.

All above methods were performed in accordance with the relevant guidelines and regulations. All data analyses were conducted in the Stata 14.1 (StataCorp LP, Texas City, USA) for MAC.

## Results

### Characteristics of the participants

As shown in Table 1, of all participants, 69.7% were female, 71.1% were younger than 39 years, 75.4% were married or ever married, 30.8% had attained an education of bachelor’s degree or above, 69.0% held a primary technical title, 43.5% received an income of 2000 Yuan or below per month, 22.0% had an administrative duty, 73.2% were working in county-level healthcare institutions. Significant differences were observed between RHWs with and without the experience of job mobility with respect to gender, age, marriage, education, and technical title. Compared with RHWs without the experience of job mobility, these with such experience presented a significantly higher percentage in the following groups: female, ≥40 years, married, medical college’s education, and intermediate technical title.

**Table 1:** Socio-demographic characteristics of participants

Characteristics	Total, ***n*** (***%***)	Experience of job mobility		***p-***value ^†^
		No, ***n*** (***%***)	Yes, ***n*** (***%***)	
Gender (*n* = 3753)				0.023
Female	2616 (69.7)	1376 (52.6)	1240 (47.4)	
Male	1137 (30.3)	639 (56.2)	498 (43.8)	
Age (*n* = 3710)				< 0.001
≤ 29 years	1306 (35.2)	809 (61.9)	497 (38.1)	
30–39 years	1332 (35.9)	675 (50.7)	657 (49.3)	
≥ 40 years	1072 (28.9)	508 (47.4)	564 (52.6)	
Marriage (*n* = 3754)				< 0.001
Unmarried	924 (24.6)	576 (62.3)	348 (37.7)	
Married	2830 (75.4)	1438 (50.8)	1392 (49.2)	
Education (*n* = 3771)				< 0.001
≤ Technical secondary school	780 (20.7)	413 (52.9)	367 (47.1)	
Medical college	1829 (48.5)	934 (51.1)	895 (48.9)	
≥ Bachelor’s degree	1162 (30.8)	678 (58.3)	484 (41.7)	
Technical title (*n* = 3698)				< 0.001
Primary	2553 (69.0)	1409 (55.2)	1144 (44.8)	
Intermediate	838 (22.7)	397 (47.4)	441 (52.6)	
Senior	307 (8.3)	169 (55.0)	138 (45.0)	
Income per month (*n* = 3599)				0.741
≤ 2000 Yuan	1567 (43.5)	849 (54.2)	718 (45.8)	
2001–3000 Yuan	1398 (38.8)	751 (53.7)	647 (46.3)	
≥ 3001 Yuan	634 (17.6)	332 (52.4)	302 (47.6)	
Administrative duty (*n* = 3783)				0.516
No	2950 (78.0)	1583 (53.7)	1367 (46.3)	
Yes	833 (22.0)	447 (53.7)	386 (46.3)	
Type of profession (*n* = 3783)				0.180
Doctor	2245 (59.3)	1219 (54.3)	1026 (45.7)	
Nurse	1538 (40.7)	811 (52.7)	727 (47.3)	
Type of healthcare institution (*n* = 3783)				0.250
Township healthcare center	1015 (26.8)	535 (52.7)	480 (47.3)	
County-level institution	2768 (73.2)	1495 (54.0)	1273 (46.0)	

*Note.* ^†^ Pearson’s chi-squared test

### Job mobility and work commitment of the participants

As shown in Tables 2, 46.3% of all participants had the experience of job mobility in the past. Of them, with regard to the time of the last job mobility, 37.4 and 14.3% were in the last 3 years and in the last four to 5 years, respectively. In terms of the type of the last job mobility, 33.4, 36.5, and 17.7% moved to current institution from a healthcare institution at the same hierarchical level, from a healthcare institution at a lower hierarchical level, and from a healthcare institution at a higher hierarchical level, respectively; besides, 12.4% moved from a non-healthcare institution to current institution.

**Table 2:** Experience of job mobility and work commitment of participants

Characteristics	Total, ***n*** (***%***)	Work commitment			
		Pride in work, mean ± SD	Concern for work, mean ± SD	Dedication to work, mean ± SD	Having a turnover intent, ***n*** (***%***)
Overall		3.54 ± 0.85	3.81 ± 0.81	3.61 ± 0.87	1132 (29.9)
Experience of job mobility (*n* = 3783)					
No	2030 (53.7)	3.51 ± 0.86	3.78 ± 0.80	3.58 ± 0.87	580 (28.6)
Yes	1753 (46.3)	3.58 ± 0.84	3.84 ± 0.81	3.64 ± 0.86	552 (31.5)
*p-*value		0.020 ^a^	0.025 ^a^	0.059 ^a^	0.028 ^b^
Time of the last job mobility (*n* = 1641)					
≤ 3 years	614 (37.4)	3.66 ± 0.82	3.90 ± 0.81	3.66 ± 0.86	184 (30.0)
4–5 years	234 (14.3)	3.43 ± 0.87	3.70 ± 0.85	3.52 ± 0.83	93 (39.7)
≥ 6 years	793 (48.3)	3.57 ± 0.84	3.85 ± 0.79	3.66 ± 0.87	239 (30.1)
*p-*value		< 0.001 ^a^	0.001 ^a^	0.034 ^a^	0.006 ^b^
Type of the last job mobility (*n* = 1723)					
Lateral mobility	575 (33.4)	3.63 ± 0.80	3.87 ± 0.80	3.67 ± 0.86	181 (31.5)
Upward mobility	629 (36.5)	3.59 ± 0.88	3.83 ± 0.84	3.66 ± 0.87	181 (28.8)
Downward mobility	305 (17.7)	3.50 ± 0.85	3.84 ± 0.78	3.57 ± 0.84	113 (37.0)
Other mobility	214 (12.4)	3.50 ± 0.81	3.80 ± 0.78	3.56 ± 0.83	68 (31.8)
*p-*value		0.015 ^a^	0.134 ^a^	0.094 ^a^	0.032 ^b^

*Note.* ^a^ One-way analysis of variance. ^b^ Pearson’s chi-squared test. The crosstab results between time & type of the last job mobility and work commitment were presented in Table 1 Additional file 1

In terms of participants’ work commitment, the mean scores of pride in, concern for, and dedication to work were 3.54 ± 0.85, 3.81 ± 0.81, and 3.61 ± 0.87, out of a maximum of 5, respectively, and 29.9% of participants reported a turnover intent. Significant differences were observed between RHWs with and without the experience of job mobility with respect to their pride in and concern for work and turnover intent.

### Multivariate regression analyses

Table 3 presents the results of multivariate regression equations. With regard to the overall experience of job mobility in the past, after controlling for the effects of the covariates, only the relationship between turnover intent and experience of job mobility was significant. In other words, RHWs having experienced the job mobility in the past were 1.19 times (95% CI: 1.02–1.38) more likely to present a turnover intent toward their current healthcare institutions. These results, therefore, provide limited support for Hypothesis 1.

**Table 3:** Multivariate analysis of the association between job mobility and work commitment of participants

Dependent variables	Pride in Work	Concern for work	Dedication to work	Turnover intent (0 = no, 1 = yes)
**Block I**				
Experience of job mobility (ref. = no)				
Yes	0.04 (1.22)	0.04 (1.37)	0.04 (1.39)	1.19 (1.02, 1.38) ^*^
**Block II**				
Time of the last job mobility (ref. = no)				
≤ 3 years	0.16 (3.96) ^***^	0.14 (3.70) ^***^	0.12 (2.82) ^**^	1.00 (0.81, 1.23)
4–5 years	−0.08 (−1.38)	− 0.09 (− 1.56)	−0.04 (− 0.67)	1.54 (1.15, 2.07) ^***^
≥ 6 years	− 0.03 (− 0.73)	−0.01 (− 0.16)	< 0.01 (0.06)	1.23 (1.01, 1.51) ^*^
**Block III**				
Type of the last job mobility (ref. = no)				
Lateral mobility	0.11 (2.63) ^**^	0.10 (2.41) ^*^	0.07 (1.59)	1.15 (0.93, 1.42)
Upward mobility	0.03 (0.74)	−0.01 (− 0.13)	0.07 (1.54)	1.13 (0.91, 1.40)
Downward mobility	−0.01 (− 0.18)	0.06 (1.19)	− 0.01 (− 0.14)	1.32 (1.00, 1.74) ^*^
Other mobility	− 0.06 (− 0.98)	−0.01 (− 0.07)	−0.02 (− 0.30)	1.28 (0.93, 1.77)
**Block IV**				
Time & type of the last job mobility (ref. = no)				
≤ 3 years & lateral	0.24 (3.88) ^***^	0.20 (3.36) ^***^	0.16 (2.47) ^*^	0.96 (0.69, 1.32)
≤ 3 years & upward	0.17 (2.52) ^*^	0.15 (2.31) ^*^	0.21 (3.01) ^**^	0.94 (0.66, 1.34)
≤ 3 years & downward	0.07 (0.92)	0.10 (1.32)	0.01 (0.13)	1.10 (0.74, 1.63)
≤ 3 years & other	0.10 (0.77)	0.11 (0.94)	−0.04 (− 0.33)	1.03 (0.55, 1.91)
4–5 years & lateral	0.01 (0.05)	<−0.01 (− 0.02)	−0.01 (− 0.13)	2.00 (1.24, 3.23) ^**^
4–5 years & upward	− 0.02 (− 0.19)	−0.15 (− 1.46)	0.01 (0.04)	1.07 (0.62, 1.84)
4–5 years & downward	− 0.09 (− 0.74)	− 0.04 (− 0.38)	−0.08 (− 0.71)	1.43 (0.82, 2.48)
4–5 years & other	−0.51 (− 2.86) ^**^	−0.37 (− 2.21) ^*^	−0.13 (− 0.71)	2.48 (1.07, 5.71) ^*^
≥ 6 years & lateral	0.01 (0.10)	< 0.01 (0.05)	−0.03 (− 0.42)	1.05 (0.77, 1.45)
≥ 6 years & upward	− 0.03 (− 0.60)	−0.05 (− 1.03)	0.04 (0.52)	1.30 (0.99, 1.71)
≥ 6 years & downward	−0.11 (− 1.06)	0.08 (0.82)	− 0.03 (− 0.31)	2.05 (1.24, 3.37) ^**^
≥ 6 years & other	−0.02 (− 0.26)	0.07 (0.88)	< 0.01 (0.03)	0.97 (0.61, 1.56)

*Note.* All sociodemographic variables were controlled in each block (Full results were presented in Table 2 in Additional file 1). The numbers in the columns 2–4 are *b* (regression coefficient) and *t* value of *b*. The numbers in the column 5 are OR and 95% CI. ^***^ *p*-value < 0.001, ^**^ *p*-value < 0.01, ^*^ *p*-value < 0.05

With respect to the time of the last job mobility, RHWs whose job changes were in the last 3 years reported significantly high levels of pride in (*b*: 0.16, *p*-value < 0.001), concern for (*b*: 0.14, *p*-value < 0.001), and dedication to work (*b*: 0.12, *p*-value < 0.01); however, job changes in the last four to 5 years (OR: 1.54, 95% CI: 1.15–2.07) and 6 years ago or more (OR: 1.23, 95% CI: 1.01–1.51) were significantly related to a higher odds of having a turnover intent. Based on above, Hypotheses 2a and 2b were supported.

In terms of the type of the last job mobility, the lateral mobility was significantly associated with a high level of pride in work (*b*: 0.11, *p*-value < 0.01) and a high level of concern for work (*b*: 0.10, *p*-value < 0.05); however, the downward mobility significantly increased the odds of having a turnover intent towards current institution (OR: 1.32, 95% CI: 1.00–1.74). These results provide support for Hypothesis 3.

Results in Block IV further show the significant interactions between different times and different types of RHWs’ last job mobilities.

## Discussion

The study reported that 46.3% of RHWs in western China had the experience of job mobility in the past, which indicated that the job change was common among RHWs. Consistent with results in our previous study [7], the work commitment of RHWs in western China was not very high. Similarly, Labrague et al. reported the moderate work commitment among rural nurses in the Philippines [5].

Overall, the results suggest that the extent of the relationship between job mobility and work commitment depends upon the dimension of commitment being measured (after accounting for the covariates). Limited support was found for Hypothesis 1 in that having experienced external job mobilities in the past was significantly related to an increased odds of having a turnover intent, i.e., a low-level work commitment. This means that RHWs’ overall experience of job mobility could be considered as a threat to their work commitment, which is consistent with results reported by Kondratuk et al. [16] and Murrell et al. [19] and provides evidence to a great deal of previous discussion about the potential decline in the commitment to organizations due to the increase in job mobility [15]. A potential explanation is that these RHWs who had experienced job mobilities in the past could be considered to have a “careerist attitude”. This careerism perspective was defined as “the propensity to pursue career advancement through non-performance-based means,” which included career mobility tactics and the instrumental use of social relationships with co-workers, supervisors, etc. [20] Individuals with a careerist attitude might be highly sensitive to the negative aspects of their job and the organizations in which they worked [21]. In general, these RHWs having experienced job mobilities in the past might be more committed to themselves rather than the healthcare institutions where they worked.

In addition to the overall experience of job mobility, we also analyzed the association between RHW’s last job mobilities and their work commitment. The results suggest that the extent of the relationship between the last job mobility and work commitment depends upon the dimension of commitment being measured (after adjusting for the covariates) and the time (≤3 years versus 4–5 years versus ≥6 years) of mobility and the type (lateral versus upward versus downward versus other). We found the different associations of work commitment with different types of RHWs’ last job mobilities. For example, the last job changes between two healthcare institutions at the same hierarchical level (i.e., lateral mobility) were significantly associated with a high level of pride in and concern for work; however, the downward mobilities (i.e., job changes from a healthcare institution at a higher hierarchical level to current institution) were related to a higher odds of having a turnover intent. These differences are expected as different types of job mobilities would lead to different changes in RHWs’ working and living conditions. Nevertheless, generalizations concerning the effects of RHWs’ last job mobilities of different types should be avoided. Results of the interaction between different types and different times of the last job mobilities on their association with the work commitment suggest that the time of the last job mobility was an important moderator.

In terms of the time of the last job mobility, in line with the prior literature [14, 16, 17], we found that the last job mobility that occurred over relatively recent periods of time was related positively to work commitment. In our study, compared with RHWs without experience of job mobility in the past, those whose last job changes were in the last 3 years reported a significantly higher level of work commitment, i.e., pride in, concern for, and dedication to work. However, as time went on after the last job mobility of RHWs, they presented a significantly lower level of work commitment, i.e., an increased odds of having the turnover intent, than those RHWs without the experience of job mobility. Kalleberg et al. have reported similar findings [18]. Although we didn’t find any studies related to the association between job mobility and work commitment among healthcare workers, let alone RHWs, some reasons reported in related studies from other industries may explain these findings.

For RHWs whose last job changes were in the last 3 years, especially these lateral (i.e., job change between two different healthcare institutions at the same hierarchical level) or upward (i.e., job change from a healthcare institution at a lower hierarchical level to current institution) mobilities, the new job may be considered as a positive contrast to the former professional situation in terms of better working conditions, improvement in quality of life, and a workplace that better meets their development and advancement needs, for example [36]. Therefore, these RHWs reported a high level of work commitment, which could be the consequences of leaving a healthcare institution and switching from an unsatisfactory situation to a better one [14]. Besides, the significant positive associations of lateral or upward mobilities in the last 3 years with a high-level work commitment could be the consequences of entering a new healthcare institution with all the excitingly new aspects that this entails [14]. A honeymoon effect therefore may explain our results that refer to the trend whereby work commitment decreases substantially before job mobility and increase after it [27, 28, 37]; moreover, the results may be affected the cognitive dissonance, i.e., RHWs who have changed their jobs may tend to legitimize the job changes by reporting a more positive description of the new job in the initial period of time [17]. However, the positive effects of job change may disappear with adaption and normalization taking place for a while after the job mobility, and work commitment returns progressively to its initial level or even lower, which could be called the hangover effect [14, 17, 27]. In our study, we did not continue to identify the significant associations of the lateral and upward mobilities 4 years ago or more with their pride in, concern for, and dedication to work, and although not significant, many RHWs whose last job changes were lateral or upward mobilities 4 years ago or more reported a low level of pride in, concern for, and dedication to work. Meanwhile, RHWs whose last job changes were lateral mobilities in the last four to 5 years were 2.00 times more likely to have the turnover intent compared with those without experience of job changes. Besides, the last job changes as downward mobilities six years ago or more or as other mobilities in the last four to five years were also significantly related to a low level of work commitment. In addition, selection biases might provide an alternative interpretation for the observed relationship between last job changes 4 years ago or more and the work commitment. For example, if many career-oriented RHWs (with above-average commitment) quitted their jobs after 3 years to start a different job, the remaining workers had relatively little commitment in comparison; in consequence, it may come to an overrepresentation of more negative cases in this group of RHWs who reported mobility in the past.

In sum, it should be stressed that job mobility is often considered as a threat [14]; however, job mobility may offer to individuals of RHWs more gains than costs in the initial period after the job change [14, 19], such as RHWs within 3 years of changing jobs as lateral or upward mobilities, and even RHWs within 3 years of changing jobs as downward or other mobilities reported higher average scores of pride in and concern for work than those of RHWs without any experience of job changes. As Boswell et al. argued, these results are not surprising, and job mobility may stimulate positive attitudes in employees regardless of the reason for the job change because they need to make sense of a new job [27]. However, when 4 years or more have passed after job changes, it seems that job mobility becomes a threat to the work commitment of RHWs, especially for those whose last job changes are lateral, downward, and other mobilities. Therefore, as discussed before, we need to depict the association between job mobility and work commitment of RHWs systematically, and identify these RHWs who have the experience of job mobility related to a low level of work commitment.

Several limitations of our study should be addressed. First, some caution is warranted in interpreting the results because measures were self-reported. Some answers were retrospective, so recall biases can therefore occur. Second, this study mainly focused on the affective commitment of RHWs and could not provide an overall depiction of the work commitment and its association with the job mobility of RHWs. Third, although the study reported the time and type of the last job mobility of RHWs, it did not look at whether RHWs’ last job mobilities were voluntary or involuntary and promoted or demoted, which might also be important aspects related to the association between job mobility and work commitment of RHWs. Fourth, although we controlled for nine sociodemographic variables of RHWs in the multivariate analyses, some other variables were missing; for example, the status of employment, i.e., permanent or temporary position, was usually a very important controlling factor in past studies on RHWs. Fifth, the sample size of RHWs that was set by the PI, all co-PIs, and two WHO experts was not a very scientific measure, hence the potential bias of the sample representativeness in our study. Meanwhile, as certain types of RHWs with shorter or longer years in current job might be more or less likely to take part in the survey, these selection biases of participants might exist in the study and could affect the results. Sixth, as the study was conducted in western China, some findings might not apply well to the RHWs working in other regions of China. Seventh, as the survey was conducted in 2013, the data are somewhat out of date and it cannot provide the most recent research findings. Finally, because of the limitations of our study design, the causal relationship between job mobility of RHWs and their work commitment could not be concluded. A quasi-experimental approach or other appropriate study designs could be employed in our future research to identify the causal evidence for the effect of job mobility on the work commitment of RHWs.

## Conclusions

This is the first study in China to analyze the association of job mobility with work commitment among RHWs. Job change was common and work commitment was not very high for RHWs. The overall experience of job mobility in the past of RHWs was significantly associated with a low level of work commitment of RHWs (i.e., an increased odds of having a turnover intent). With respect to the last job mobility, a honeymoon-hangover pattern was found among its association with RHWs’ work commitment. Specifically, RHWs whose last job changes were in the last 3 years, especially these lateral and upward mobilities, reported a significantly high level of pride in, concern for, and dedication to work. However, the last job changes as lateral mobilities in the last four to 5 years, downward mobilities 6 years ago or more, and other mobilities in the last four to 5 years were significantly associated with an increased odds of having the turnover intent, and the last job changes as other mobilities in the last four to 5 years were also related to a low level of pride in and concern for work. These results suggest that job change may help RHWs to be more committed to their new work in the initial period after job mobility; as time pass by, for example, 4 years later, RHWs would be less committed.

From the perspective of rural healthcare workforce management, there are several practical implications of our findings. The managers of rural healthcare institutions should pay more attention to RHWs with the experience of job mobility in the past to improve their work commitment. More research is needed to identify the factors that are positively related to the work commitment of RHWs, especially of these having experienced job mobilities in the past, and after that, rural healthcare institutions can implement some targeted strategies. Meanwhile, it’s important to note that, researchers in psychology and sociology have powerfully documented that expectations can trigger self-fulfilling processes [15, 38, 39], which means that if the managers reduce their commitment to RHWs with the experience of job mobility as who are perceived to be less committed, it would cause these RHWs to indeed become less committed. Thus, the managers should be positively involved in rural healthcare workforce management. In addition, our study provides evidence on when the hangover of a RHW after the job change is likely to begin, and there appear to be “risky periods” in which RHWs are likely to experience declining work commitment. The managers of rural healthcare institutions should consider timing when interpreting RHWs’ affective reactions toward their work; for example, they would expect a high work commitment level among RHWs initially but should not be surprised (or necessarily alarmed) to see this decline over time. Rural healthcare institutions could educate newcomers of RHWs on the expected pattern of work commitment as part of their on-boarding process and realistic job previews. It may also informative should the managers not observe the honeymoon-hangover pattern. For example, relatively flat levels of work commitment (i.e., no honeymoon period) may suggest that RHWs’ early experience related to the fulfilled commitments are lacking; conversely, to the extent that work commitment continues to decline, this may signal a need to intervene particularly for those RHWs that the rural healthcare institution hopes most to retain.

## Supplementary Information

**Additional file 1.** Additional analysis results of job mobility and work commitment of RHWs.

## Data Availability

Data are available upon reasonable request from the corresponding author.

## Abbreviations

SDGs: Sustainable Development Goals
WHO: World Health Organization
RHWs: Rural healthcare workers
PI: Principal investigator
THC: Township healthcare center
CDC: Center for disease control and prevention
TCMH: Traditional Chinese medical hospital
MCHH: Maternity and child healthcare hospital
CGH: County general hospital
CFA: Confirmatory factor analysis
ANOVA: Analysis of variance
OR: Odds ratio
95% CI: 95% confidential interval
