# Supplementary material for: How does job mobility relate to work commitment among rural healthcare workers? a cross-sectional study in western China
Source: BMC Health Serv Res. 2021 Oct 20;21:1126. doi: 10.1186/s12913-021-07166-w (PMC8527660; doi:10.1186/s12913-021-07166-w)
Supplement: Supplementary file 1 — Additional file 1. Additional analysis results of job mobility and work commitment of RHWs. [file 12913_2021_7166_MOESM1_ESM.docx]

**Table 1** Time & type of the job mobility and work commitment of participants

| **Characteristics** | **Total, *n* (*%*)** | **Work commitment** | | | |
| --- | --- | --- | --- | --- | --- |
|  |  | **Pride in work, Mean**±**SD** | **Concern for work, Mean**±**SD** | **Dedication to work, Mean**±**SD** | **Having a turnover intent, *n* (*%*)** |
| Time & type of the last job mobility (*n*=1611) | | | | | |
| ≤3 years & lateral | 227 (14.1) | 3.72±0.76 | 3.93±0.80 | 3.69±0.81 | 66 (29.1) |
| ≤3 years & upward | 179 (11.1) | 3.70±0.89 | 3.94±0.84 | 3.79±0.90 | 49 (27.4) |
| ≤3 years & downward | 147 (9.1) | 3.55±0.83 | 3.83±0.77 | 3.54±0.85 | 52 (35.4) |
| ≤3 years & other | 56 (3.5) | 3.62±0.79 | 3.89±0.81 | 3.47±0.89 | 15 (26.8) |
| 4−5 years & lateral | 74 (4.6) | 3.50±0.84 | 3.77±0.82 | 3.56±0.84 | 35 (47.3) |
| 4−5 years & upward | 69 (4.3) | 3.51±0.92 | 3.66±0.93 | 3.54±0.83 | 20 (29.0) |
| 4−5 years & downward | 58 (3.6) | 3.39±0.84 | 3.70±0.81 | 3.48±0.77 | 23 (39.7) |
| 4−5 years & other | 27 (1.7) | 3.11±0.88 | 3.48±0.91 | 3.46±0.98 | 14 (51.9) |
| ≥6 years & lateral | 240 (14.9) | 3.57±0.80 | 3.82±0.78 | 3.63±0.91 | 67 (27.9) |
| ≥6 years & upward | 353 (21.9) | 3.58±0.87 | 3.82±0.82 | 3.67±0.86 | 107 (30.3) |
| ≥6 years & downward | 76 (4.7) | 3.50±0.90 | 3.96±0.78 | 3.66±0.88 | 33 (43.4) |
| ≥6 years & other | 105 (6.5) | 3.59±0.82 | 3.93±0.74 | 3.66±0.82 | 26 (24.8) |
| *p-*value | | 0.003 ^†^ | 0.012 ^†^ | 0.093 ^†^ | <0.001 ^‡^ |

*Note.* ^†^ One-way analysis of variance. ^‡^ Pearson’s chi-squared test.

**Table 2** Multivariate analysis of the association between job mobility and work commitment of participants

| **Dependent variables** | **Pride in**  **Work** | **Concern for work** | **Dedication to work** | **Turnover intent**  **(0=no, 1=yes)** |
| --- | --- | --- | --- | --- |
| **Block I** |  |  |  |  |
| Experience of job mobility (ref.=no) | | | | |
| Yes | 0.04 (1.22) | 0.04 (1.37) | 0.04 (1.39) | 1.19 (1.02, 1.38) ^*^ |
| Gender (ref.=female) | | | | |
| Male | −0.04 (−1.02) | −0.02 (−0.69) | 0.06 (1.63) | 0.98 (0.81, 1.18) |
| Age (ref.=≤29 years) | | | | |
| 30−39 years | 0.02 (0.53) | 0.02 (0.43) | 0.02 (0.34) | 1.04 (0.85, 1.30) |
| ≥40 years | 0.06 (1.05) | 0.002 (0.04) | 0.06 (1.17) | 0.74 (0.57, 0.98) ^*^ |
| Marriage (ref.=unmarried) | | | | |
| Married | 0.02 (0.48) | 0.07 (1.81) | 0.03 (0.73) | 1.04 (0.83, 1.29) |
| Education (ref.=≤technical secondary school) | | | | |
| Medical college | −0.05 (−1.27) | −0.001 (−0.02) | 0.05 (1.37) | 1.18 (0.97, 0.45) |
| ≥Bachelor’s degree | −0.13 (−2.84) ^**^ | −0.01 (−0.24) | 0.01 (0.15) | 1.39 (1.10, 1.77) ^**^ |
| Technical title (ref.=primary) | | | | |
| Intermediate | −0.04 (−0.83) | −0.001 (−0.03) | 0.004 (0.10) | 1.11 (0.89, 1.37) |
| Senior | 0.08 (1.31) | 0.04 (0.61) | 0.08 (1.29) | 1.08 (0.78, 1.49) |
| Income per month (ref.=≤2000 Yuan) | | | | |
| 2001−3000 Yuan | 0.12 (3.46) ^***^ | 0.12 (3.67) ^***^ | 0.13 (3.69) ^***^ | 0.81 (0.68, 0.96) ^*^ |
| ≥3001 Yuan | 0.17 (3.67) ^***^ | 0.14 (3.31) ^***^ | 0.04 (0.87) | 0.59 (0.46, 0.75) ^***^ |
| Administrative duty (ref.=no) | | | | |
| Yes | 0.18 (4.64) ^***^ | 0.21 (5.93) ^***^ | 0.20 (5.26) ^***^ | 1.11 (0.92, 1.35) |
| Type of profession (ref.=doctor) | | | | |
| Nurse | 0.04 (0.99) | 0.01 (0.25) | 0.02 (0.61) | 0.81 (0.67, 0.98) ^*^ |
| Type of healthcare institution (ref.=township healthcare center) | | | | |
| County-level institution | −0.01 (−0.17) | −0.03 (−0.79) | −0.08 (−2.17) | 0.85 (0.71, 1.00) |

*Note.* The numbers in the columns 2−4 are *b* (regression coefficient) and *t* value of *b*. The numbers in the column 5 are OR and 95% CI. ^***^ *p*-value <0.001, ^**^ *p*-value <0.01, ^*^ *p*-value <0.05.
